# A Lipid Receptor Sorts Polyomavirus from the Endolysosome to the Endoplasmic Reticulum to Cause Infection

**DOI:** 10.1371/journal.ppat.1000465

**Published:** 2009-06-05

**Authors:** Mengding Qian, Dawen Cai, Kristen J. Verhey, Billy Tsai

**Affiliations:** 1 Department of Cell and Developmental Biology, University of Michigan Medical School, Ann Arbor, Michigan, United States of America; 2 Department of Molecular and Cellular Biology, Harvard University, Cambridge, Massachusetts, United States of America; Fred Hutchinson Cancer Research Center, United States of America

## Abstract

The mechanisms by which receptors guide intracellular virus transport are poorly characterized. The murine polyomavirus (Py) binds to the lipid receptor ganglioside GD1a and traffics to the endoplasmic reticulum (ER) where it enters the cytosol and then the nucleus to initiate infection. How Py reaches the ER is unclear. We show that Py is transported initially to the endolysosome where the low pH imparts a conformational change that enhances its subsequent ER-to-cytosol membrane penetration. GD1a stimulates not viral binding or entry, but rather sorting of Py from late endosomes and/or lysosomes to the ER, suggesting that GD1a binding is responsible for ER targeting. Consistent with this, an artificial particle coated with a GD1a antibody is transported to the ER. Our results provide a rationale for transport of Py through the endolysosome, demonstrate a novel endolysosome-to-ER transport pathway that is regulated by a lipid, and implicate ganglioside binding as a general ER targeting mechanism.

## Author Summary

To cause infection, viruses must reach appropriate compartments within the cell where they undergo a programmed series of conformational changes that enable the viral genome to be exposed and released. The mechanisms that target viruses to these compartments are often not clear. Here we study the infectious pathway of the murine polyomavirus (Py). Py is transported from the cell surface to the intracellular organelle called the endoplasmic reticulum (ER), where it breaches the ER membrane to reach the nucleus to stimulate infection. How Py is transported from the cell surface to the ER is poorly characterized. Our studies show that Py first enters the endolysosome compartments before reaching the ER. The low pH of the endolysosome imparts a structural change on the virus that facilitates its subsequent ER membrane penetration. Importantly, transport of Py from the endolysosome to the ER is guided by the lipid receptor ganglioside GD1a. We also demonstrate that an artificial bead capable of binding to GD1a is transported to the ER as well. Collectively, our data identify a lipid-dependent mechanism that targets a virus to its appropriate organelle during infection.

## Introduction

Viruses must navigate through the complex endocytic machineries of the host cell to successfully cause infection. Although some viruses evade or escape degradative compartments such as the endolysosome to infect cells, others rely on this organelle to facilitate infection [1]. How these processes are regulated is poorly understood.

The non-enveloped murine polyomavirus (Py) is transported from the cell surface to the nucleus where transcription and replication of the viral DNA genome lead to lytic infection and cell transformation. The successful arrival of one viral particle to the nucleus is sufficient to cause infection [2]. Py is composed of 72 pentamers of the outer structural protein VP1, which associate with the internal proteins VP2 and VP3 and encapsulate the DNA genome [3].

To initiate infection, VP1 binds to the glycolipid receptor ganglioside GD1a on the plasma membrane and is transported to the lumen of the endoplasmic reticulum (ER) [4]. Transport to the ER is essential for infection as inactivation of ER-resident factors blocks infection significantly [5]–[7]. Py then penetrates the ER membrane, likely enabling it to reach the cytosol and then the nucleus. The precise mechanism controlling the transport of Py from the plasma membrane to the ER, a decisive step in the virus entry pathway, remains to be clarified.

Other members of the polyomavirus family including SV40 and the human BK virus also bind to ganglioside glycolipids [4], [8]–[10]. This is in contrast to many viruses that rely on glycoproteins as entry receptors [1],[11]. Upon internalization, most gangliosides are transported to the early and late endosomes, reaching the lysosome where they are eventually hydrolyzed by lysosomal enzymes. Although a small fraction of gangliosides can reach the Golgi from the plasma membrane, their retrograde transport to the ER has not been observed [12].

Using a combination of live cell fluorescence microscopy, biochemistry, and cell infection studies, we show that Py is transported to the endolysosome and that the low pH environment is critical for infection. Strikingly, we find that GD1a sorts Py from the endolysosome to the ER. Binding to GD1a is likely the key event to direct Py to the ER as an artificial particle coated with GD1a antibody binds to GD1a and is transported to the ER. Our results provide an explanation for trafficking of Py through the endolysosome, demonstrate an endolysosome-to-ER transport pathway that is controlled by a lipid, and implicate ganglioside binding as a general ER targeting mechanism.

## Results

### Live cell imaging of polyomavirus transport to the endolysosome

**Figure 1:**
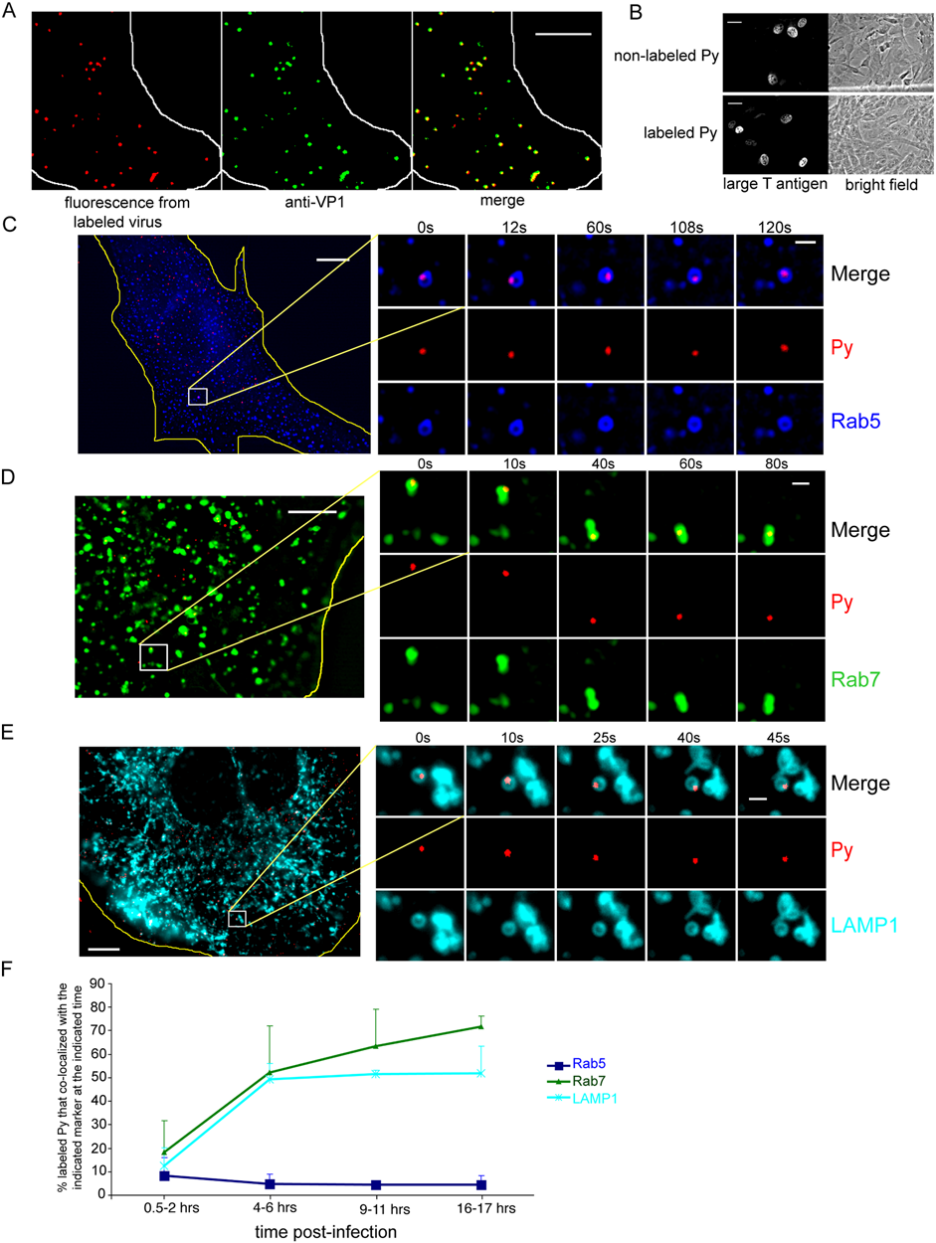
Time-dependent transport of Py through the endolysosome. (A) NIH 3T3 cells were incubated with Texas-Red-labeled purified Py for 3 hrs at 37°C, washed, and subjected to immunofluorescence using a VP1 antibody followed by FITC-labeled secondary antibody. White line, edge of cell. Scale bar, 5 µm. (B) Cells were incubated with labeled or non-labeled Py for 48 hrs, and the extent of infection assessed by immunofluorescence using a Py large T antigen antibody. Cells stained with large T antigen in the nucleus were scored as positive for infection. Scale bars, 30 µm. (C–E) Live cell imaging of labeled Py in cells expressing (C) CFP-Rab5, (D) YFP-Rab7, or (E) LAMP1-YFP. C, D, and E are images taken at the 4–6 hrs post-infection time point. Yellow lines, edge of cells. Scale bars, 10 µm (whole cell) and 1 µm (inset). (F) Quantification of the extent of co-localization between labeled Py and the respective markers at the indicated post-infection time points. For each time point, at least 90 viral particles were analyzed from 3 cells. Data are the mean+/−SD.

As GD1a is normally transported through the endolysosome, we hypothesized that Py binds to this lipid molecule and is also transported initially through this pathway. To visualize the transport of Py in murine NIH 3T3 cells in real time, purified Py was labeled with the Texas-Red (or Alexa Fluor 594) dye and imaged using wide field fluorescence microscopy. We first assessed whether labeling affected virus entry and infection. We found that, at 3 hrs post-infection, the fluorescence of the labeled virus in cells overlapped significantly with the pattern seen with immunofluorescence using an antibody against VP1 (Figure 1A). This finding demonstrates that Py is labeled efficiently and that the dye does not dissociate from the virus after entry. Next, using expression of the virally-encoded large T antigen to measure infection, we found that 7.6% of cells infected with the non-labeled virus expressed large T antigen, and 7.5% of cells infected with the labeled virus expressed large T antigen (Figure 1B). This result indicates that the labeling procedure did not affect Py infection, consistent with a previous observation [13]. We conclude that the labeled Py recapitulates the normal cellular transport and infection processes of Py.

To determine the subcellular trafficking events that lead to Py infection, co-localization of labeled virus with fluorescent protein-tagged markers of the endolysosomal compartments was determined over time. To synchronize Py infection, cells were incubated with labeled virus at 4°C to enable cell surface binding, washed to remove unbound virus, and then shifted to 37°C to initiate entry. To rule out any coincident co-localization, the association of labeled virus with endolysosomal markers was tracked by live cell imaging. Only those Py particles that co-localized with vesicles for more than 30 s were counted as true co-localization.

When the localization of labeled Py was compared to cyan fluorescent protein (CFP)-Rab5, a marker of early endosomes, we found that less than 10% of the internalized Py co-localized with CFP-Rab5 at 0.5–2 hrs post-infection. A relatively low level of virus persists in the early endosome throughout the course of infection as seen at the 4–6 hrs and 16–17 hrs post-infection time points (Figure 1, C and F). The virus appeared to be located on the endosome membrane, suggesting that it remained attached to the membrane and was not been released into the lumen (Figure 1C). These results are consistent with findings in fixed cells where a minor population of Py co-localized with Rab5-containing early endosomes [14]. It should be noted that, when expressed at a moderate level, CFP-Rab5 does not alter significantly the general morphology or distribution of the EEA1-containing early endosomes (Figure S1A). This finding is consistent with a previous report demonstrating that low level expression of GFP-Rab5 does not affect the size of the early endosomes, or the kinetics of transferrin uptake and recycling [15].

To assess co-localization of Py with the late endosome, we asked whether the virus co-localized with yellow fluorescent protein (YFP)-Rab7, a marker of late endosomes. YFP-Rab7 has been used previously to study the behavior of the endogenous Rab7 protein [16], and we found that moderate YFP-Rab7 expression does not affect the general morphology and distribution of the LAMP1-containing late endosomes and lysosomes (Figure S1B). Our analysis showed that less than 20% of the internalized Py co-localized with YFP-Rab7 at 0.5–2 hrs post-infection (Figure 1, D and F). However, in contrast to early endosomes, Py gradually accumulated in late endosomes such that up to 70% of Py co-localized with YFP-Rab7 at later time points (Figure 1F). Similar results were observed when imaging labeled Py with LAMP1-YFP, a marker of late endosomes and lysosomes (Figure 1, E and F). In this case, the extent of co-localization increased from about 12% at the early time point to 50% at the later time points (Figure 1F, light blue line). The total percentage of Py that co-localized with the Rab7- and LAMP1-containing vesicles can exceed 100% because YFP-Rab7 and LAMP1-YFP are often located on the same vesicle (data not shown), consistent with a previous observation [17]. The co-localization of labeled Py with Rab7- and LAMP1-positive vesicles is in contrast to a previous result in fixed cells where no co-localization was reported for Py with Rab7- or LAMP2-containing vesicles [14]. The reason for this conflicting result is unknown, but could be due to differences in the detection method or cell type. We also found that Py does not co-localize with caveolin-1 in NIH 3T3 cells (Figure S2), in agreement with a previous finding [13]. Thus, we conclude that Py is transported through the endolysosomal pathway.

### Effects of expressing Rab5 and Rab7 mutants on polyomavirus infection

**Figure 2:**
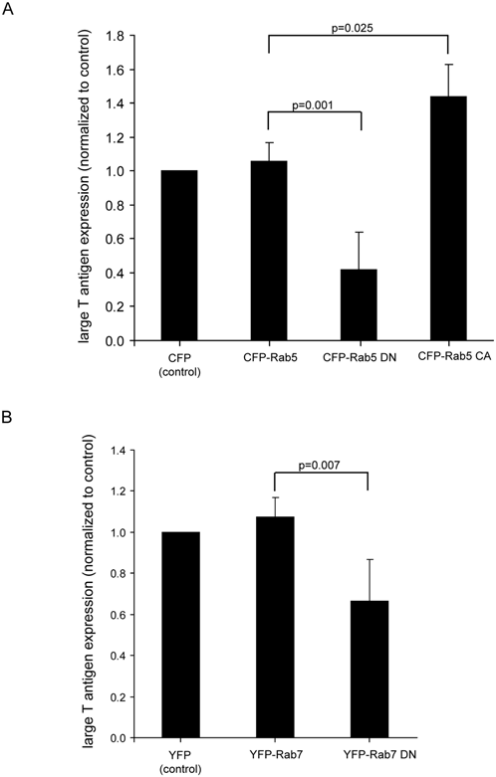
Expression of Rab5- and Rab7-interfering mutants affects Py infection. NIH 3T3 cells expressing (A) CFP control, wild-type CFP-Rab5, dominant-negative CFP-Rab5 (DN), or constitutively active CFP-Rab5 (CA) or (B) YFP control, wild-type YFP-Rab7, or dominant-negative Rab7 (DN) were incubated with Py. 48 hrs later, the extent of infection in transfected cells was assayed as in Figure 1B. Data represent the mean+/−SD of at least four independent experiments. In Figure 2A, 27/262 cells expressed T antigen in the CFP-expressing cells. In Figure 2B, 75/464 cells expressed T antigen in the YFP-expressing cells. A two-tailed *t* test was used.

As Py co-localized extensively with endolysosomal compartments, we asked if transport through these compartments is part of the Py infectious route. To do this, we took advantage of the fact that, in addition to providing organelle identity, Rab5 and Rab7 serve critical roles in regulating cargo transport within the endosomal system [18]. Accordingly, dominant-negative versions of these proteins have been shown to block cargo transport [19],[20]. We therefore tested the effect of expression of dominant-negative CFP-Rab5 S34N (CFP-Rab5 DN) on Py infection as compared to expression of either CFP (control) or wild-type CFP-Rab5. Whereas expression of CFP-Rab5 did not affect expression of Py large T antigen when compared to the control, expression of CFP-Rab5 DN caused a reduction in Py infection (Figure 2A). This result is consistent with a previous observation in which expression of dominant-negative Rab5 was reported to reduce Py infection (as data not shown) in [21]. In addition, we found that expression of a constitutively active form of Rab5, CFP-Rab5 Q79L, caused a moderate increase in Py infection (Figure 2A, CFP-Rab5 CA). We conclude that transport through the early endosome is critical for Py infection.

To assess whether transport to the late endosome plays a role in virus infection, we used YFP-Rab7 N125I (Rab7 DN), a dominant-negative Rab7 demonstrated previously to block transport of the vesicular stomatitis virus G protein from the early endosome to the late endosome [20]. Expression of YFP-Rab7 DN caused a decrease in Py infection when compared to control cells (Figure 2B), indicating that transport through the late endosomal compartment constitutes part of the Py infectious route.

### A role of the endolysosomal low pH in Py infection and conformational change

**Figure 3:**
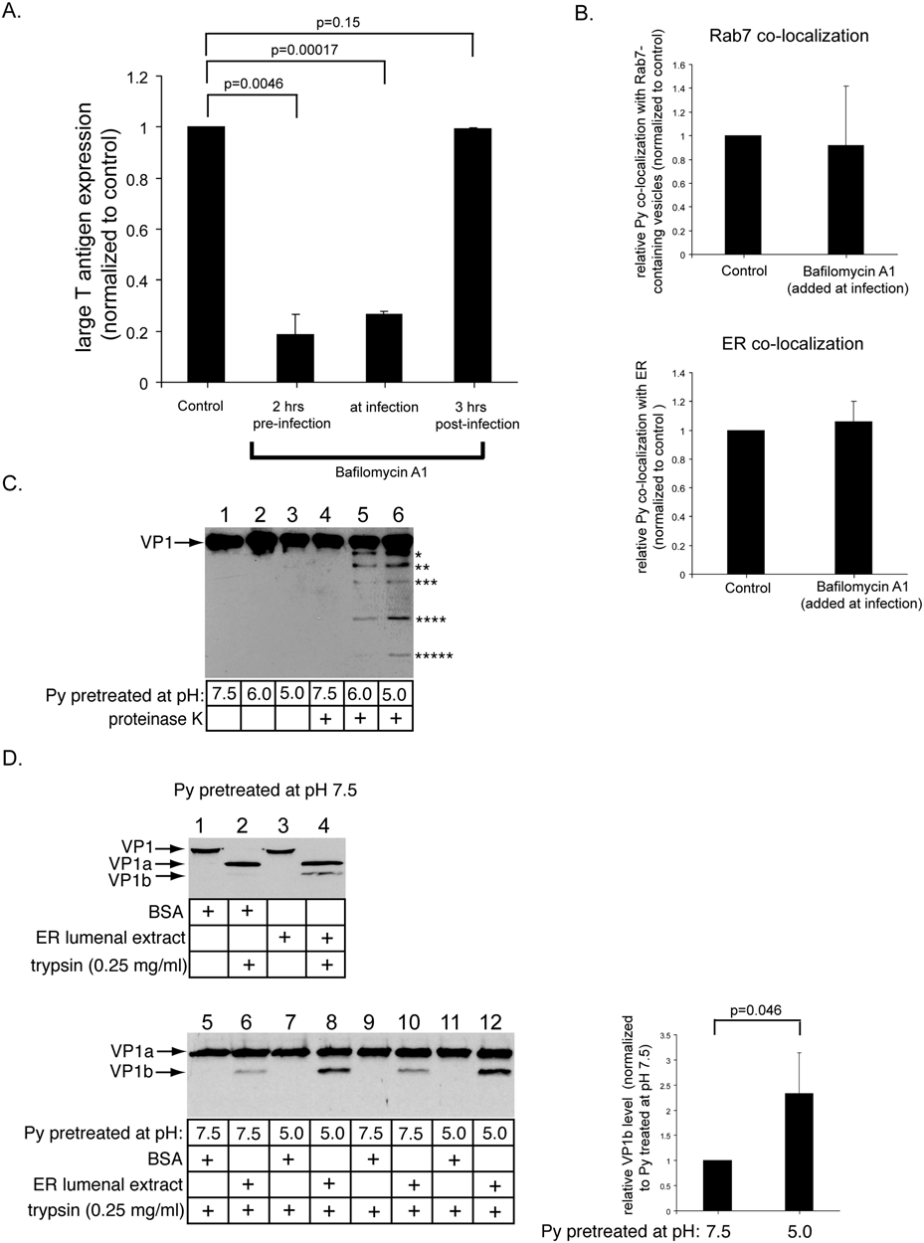
Effect of low pH on Py infection and conformational change. (A) NIH 3T3 cells were treated with bafilomycin A1 2 hrs before, at the same time, or 3 hrs after infection with non-labeled Py, and washed to remove the drug. 48 hrs later, the extent of large T antigen expression was determined as in Figure 1B. Infection efficiency was normalized to the control cells. 37/1021 cells expressed large T antigen in the control cells. A two-tailed *t* test was used. Data are the mean+/−SD. (B) Cells expressing YFP-Rab7 (top) or CFP-HO2 (bottom) were infected with non-labeled Py and treated with bafilomycin A1 simultaneously. 4.5 hrs post-infection, cells were fixed and stained with an antibody against Py VP1, followed by addition of a fluorescently tagged secondary antibody; the extent of co-localization between this fluorescent signal and the fluorescence from YFP-Rab7 or CFP-HO2 were assessed. Data are the mean+/−SD. (C) Py was incubated at pH 7.5, 6.0, or 5.0, neutralized to pH 7.5, and then treated with a low concentration of proteinase K (2.5 ng/ml). The samples were immunoblotted with an antibody against VP1. (D) Py pretreated at pH 7.5 (top and bottom panels) or at pH 5 (bottom panel) was incubated with DTT, EGTA, and either BSA or an ER lumenal extract, and then treated with a low trypsin (0.25 mg/ml) concentration. Appearance of the ER-induced VP1b fragment was analyzed by immunoblotting with an antibody against VP1. Graph on the right represents quantification of the relative VP1b level generated from Py pretreated at pH 7.5 or 5. A two-tailed *t* test was used. Data are the mean+/−SD.

Transport of Py through the endolysosomal pathway could indicate that GD1a-bound Py merely follows the ganglioside trafficking pathways. Alternatively, transit through the acidic endolysosomal pathway may be critical for the Py infectious process. To test this possibility, we asked whether the low pH in these compartments plays a role in Py infection. To this end, cells were incubated with bafilomycin A1, a specific inhibitor of the vacuolar proton ATPase that prevents acidification of endosomes, and its effect on large T antigen expression was measured. We found a significant reduction in infection in cells treated with bafilomycin A1 either 2 hrs prior to infection or at infection (Figure 3A, 2 hrs pre-infection and at infection). In contrast, when cells were treated with the drug 3 hrs after infection, no effect was seen (Figure 3A, 3 hrs post-infection). NH_4_Cl, a weak base that elevates endolysosomal pH [22], also blocks infection if it was administered either 2 hrs prior to infection or at infection, but not 3 hrs post-infection (not shown).

Perturbation of endolysosomal pH could interfere with Py infection in several ways. One possibility is that the low pH is required for cargo transport from early to late endosomes [23], such that perturbing the endosomal pH would block virus delivery to the late endosomes. This block in transport to the late endosomes would consequently interfere with delivery of the virus to the ER, a prerequisite step for infection. Alternatively, the low pH could induce conformational changes that facilitate viral penetration, as documented in other viral systems [24]. To test the first possibility, we asked whether blocking acidification of endosomes with bafilomycin A1 affects co-localization of Py with the late endosome and the ER using immunofluorescence staining. Cells expressing YFP-Rab7 were infected with non-labeled Py and treated with bafilomycin A1 simultaneously. 4.5 hrs post-infection, cells were fixed and stained with an antibody against Py VP1, followed by addition of a fluorescently tagged secondary antibody. The extent of co-localization between the anti-VP1 fluorescent signal and fluorescence from YFP-Rab7 were assessed. We found that the level of co-localization between Py and Rab7 in control and bafilomycin A1-treated cells was similar (Figure 3B, top panel), indicating that Py transport to the late endosome is not disrupted by a block in endosomal acidification. This finding is further supported by the observation that NH_4_Cl did not disrupt co-localization of Py and the Rab7-containing endosomes (not shown). In cells expressing the ER-resident protein heme oxygenase-2 fused to CFP (CFP-HO2), bafilomycin A1 did not affect co-localization of Py with the ER as well (Figure 3B, bottom panel). Collectively, these findings suggest that the endolysosomal low pH plays a critical role in facilitating Py infection, likely through acting directly on the virus. It should be noted that, while a previous finding showed that NH_4_Cl-treated cells did not block Py infection [13], another showed that it did [21].

We therefore tested the possibility that the low pH of the endolysosome imparts a conformational change in Py that facilitates its subsequent ER-to-cytosol penetration. We first determined if exposure of Py to a pH approximating the endolysosomal pH (i.e. pH 6.0 for the early endosomes and 5.0 for the late endosomes/lysosomes) induces structural changes to the virus using limited proteolysis. Py was incubated at pH 7.5, 6.0, or 5.0, neutralized to pH 7.5, and then treated with a low concentration of proteinase K (2.5 ng/ml). We found discrete fragments of VP1 were generated by this protease when Py was exposed to low pH (Figure 3C, compare lanes 5 and 6 to lane 4). In addition, Py exposed to low pH was also more sensitive to digestion with a high trypsin concentration (1 mg/ml) (Figure S3, bottom panel, compare lanes 2 and 3 to lane 1). These results indicate that low pH induces a conformational change to VP1, likely through destabilizing the virus.

We next tested whether the low pH-triggered conformational change affects an ER-dependent remodeling event critical for Py's subsequent ER-to-cytosol penetration process. Our previous work established an in vitro trypsin digestion assay that measures ER-dependent remodeling of Py required for penetration [5],[25]. Previous structural studies on Py showed that disulfide bonds and calcium ions stabilize the viral structure [3]; thus, reducing the disulfide bonds and removing the calcium ions should partially destabilize the virus. In fact, reduction of the Py disulfide bonds in the ER was shown to be dependent on PDI [7], while calcium ion extraction is likely facilitated by the ER-resident calcium-binding proteins, calnexin/calreticulin. Hence, in the in vitro assay, Py was initially incubated with the reducing agent DTT, the calcium-chelating agent EGTA, and the control protein bovine serum albumin (BSA). Under this condition, addition of a low trypsin concentration (0.25 mg/ml) resulted in appearance of a VP1-derived fragment called VP1a (Figure 3D, lane 2) [5]. In contrast, incubation of Py with an extract containing ER lumenal proteins (ER lumenal extract) instead of BSA generated an additional cleavage product called VP1b (Figure 3D, lane 4) [5]. We now find that VP1b formation generated by treatment with the ER lumenal extract was increased by pre-exposure of Py to low pH (Figure 3D, compare lane 8 to lane 6, and in duplicates, compare lane 12 to lane 10; quantified in right graph). As formation of the VP1b fragment reflects an ER-dependent conformational change that initiates ER-to-cytosol membrane penetration of Py, we conclude that the endolysosomal low pH enhances this event critical for infection.

### Decreased co-localization of Py with the late endosome and lysosome in cells supplemented with GD1a

Our results indicate that trafficking of Py through the low pH environment of endolysosomal compartments is critical for the infection processes. What is the molecular mechanism by which Py is transported through these membrane-bound compartments? The lipid receptor GD1a has been shown to stimulate infection in the ganglioside-deficient C6 cell line [4],[9], yet the step(s) in Py infection facilitated by the ganglioside are largely unknown. Thus, we asked whether GD1a promotes infection by affecting viral trafficking along the endolysosomal pathway.

**Figure 4:**
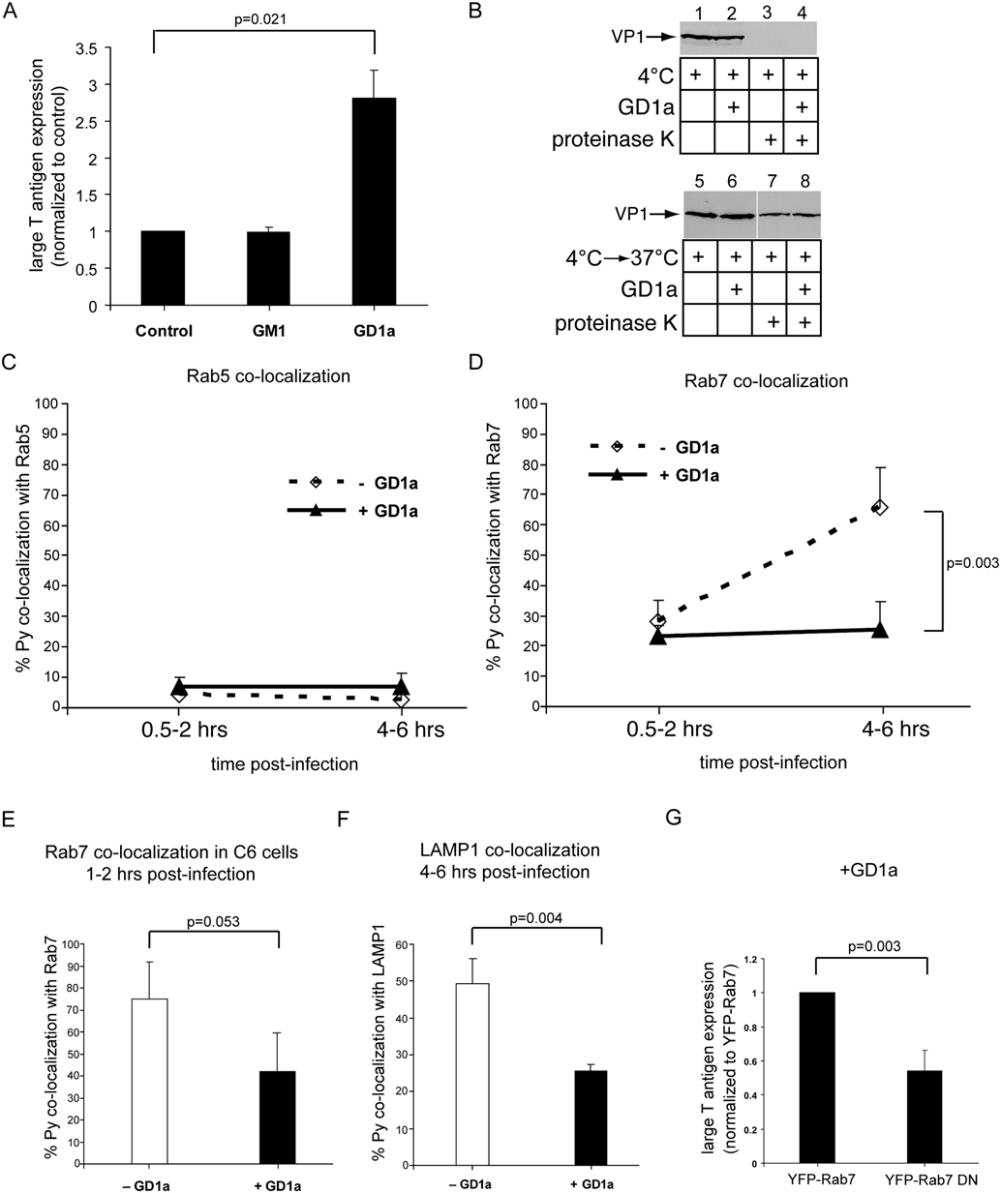
Decreased co-localization of Py with the late endosome and lysosome in GD1a-supplemented cells. (A) NIH 3T3 cells were incubated with purified GM1 or GD1a, washed, infected with Py, and the extent of infection was assessed as in Figure 1B. Results were normalized to non-supplemented cells (control cells). In the control cells, 43/984 cells expressed large T antigen. (B) Untreated (control) or GD1a-supplemented NIH 3T3 cells were incubated with Py at 4°C to allow viral binding and then treated with proteinase K where indicated (top panel) or incubated at 37°C for 1 hr before proteinase K treatment to determine viral entry (bottom panel). (C, D) The extent of co-localization of labeled Py with (C) Rab5-containing vesicles or (D) Rab7-containing vesicles at the early (0.5–2 hrs) and late (4–6 hrs) time points in both control and GD1a-supplemented NIH 3T3 cells. (E) Co-localization of labeled Py with Rab7-containing vesicles at 1–2 hrs post-infection in the ganglioside-deficient C6 cells. (F) Co-localization of labeled Py with LAMP1-containing vesicles 4–6 hrs post-infection in NIH 3T3 cells. (G) The extent of Py infection in GD1a-supplemented cells expressing wild type YFP-Rab7 or dominant negative YFP-Rab7 (DN). At least 220 transfected cells were analyzed from three independent experiments. All data are the mean+/−SD. A two-tailed *t* test was used.

We first verified that supplementing GD1a could stimulate Py infection in NIH 3T3 cells, as shown previously in C6 cells [4],[9]. Cells were incubated with either GD1a or the control ganglioside GM1 overnight, washed to remove unbound gangliosides, and then incubated with Py. At 48 hrs post-infection, cells were analyzed for large T antigen expression. As expected, Py infection was increased in cells supplemented with GD1a, but not GM1 (Figure 4A).

We then asked whether the increase in infection in GD1a-supplemented cells is due to enhanced virus binding and entry. To assess viral binding to the plasma membrane, control or GD1a-supplemented cells were incubated with Py at 4°C to allow surface binding, washed to remove the unbound virus, harvested, and the cells treated with or without proteinase K. Surface-bound virus should be sensitive to proteolysis. The proteinase K was inactivated, and the total cell lysate subjected to SDS-PAGE followed by immunoblotting with a VP1 antibody. We found a similar VP1 binding level between GD1a-supplemented and control cells (Figure 4B, compare lane 2 to 1), and the VP1 were completely sensitive to proteinase K digestion (Figure 4B, lanes 3 and 4), as expected. These results indicate that GD1a did not stimulate cell surface binding of Py, consistent with a previous result in C6 cells [9]. The lack of increased viral binding in GD1a-supplemented cells is likely due to Py VP1 binding to the sialic acid-galactose moiety present in both GD1a and glycoproteins on the cell surface [3],[4].

We next assessed whether GD1a promoted entry of Py. Cells incubated with Py at 4°C were shifted to 37°C for 1 hr to allow viral entry before treatment with proteinase K. Internalized virus should be resistant to proteinase K digestion. GD1a supplementation caused no increase in protease-resistant VP1 levels when compared to the control cells (Figure 4B, compare lane 8 to 7), suggesting that GD1a did not stimulate cell entry. We conclude that GD1a addition stimulated Py infection without increasing virus binding or entry, perhaps by facilitating transport of internalized Py to the infectious route.

We thus asked whether GD1a addition affected the co-localization of Py with early and late endosomal compartments. At both the early (0.5–2 hrs) and late (4–6 hrs) post-infection time points, GD1a addition resulted in no difference in the extent of co-localization of Py with the Rab5-containing early endosome (Figure 4C), suggesting that GD1a supplementation does not affect Py transport through early endosomal pathways. Surprisingly, a significant decrease in co-localization of Py with Rab7-containing late endosomes was observed at the 4–6 hrs post-infection time point in GD1a-supplemented cells (Figure 4D). A similar decrease in late endosomal localization upon GD1a addition was observed in C6 cells even at 1–2 hrs post-infection (Figure 4E) where a high percentage of Py (approximately 70%) already co-localized with Rab7-containing vesicles in control cells. Trafficking of Py to the late endosome in the ganglioside-deficient cells suggests that non-ganglioside receptors such as glycoproteins can mediate Py entry along the endocytic pathway. GD1a addition also caused a decrease in co-localization of Py with LAMP1-containing vesicles at the 4–6 hrs time point in NIH 3T3 cells (Figure 4F). To verify that the decrease in late endosomal localization of Py upon GD1a supplementation was not due to GD1a-induced constriction of membrane-bound compartments, we measured the size of CFP-Rab5, YFP-Rab7, or LAMP1-YFP vesicles in control or GD1a-supplemented cells and found no significant differences (Figure S4 A–C). Taken together, these results indicate that GD1a supplementation caused a decrease in the co-localization of Py with late endosome and lysosome compartments.

To exclude the possibility that addition of GD1a creates a novel infectious pathway that does not require the endolysosome, we asked whether transport through this compartment is still required for Py infection in GD1a-supplemented cells. We found that expression of YFP-Rab7 DN decreased infection in GD1a-supplemented cells (Figure 4G), similar to the results in control cells (Figure 2B). Thus, in GD1a-supplemented cells, transport through the endolysosome remains critical for infection.

That addition of GD1a to cells stimulates Py infection by decreased co-localization of Py with the late endosome and lysosome suggests that GD1a functions to sort Py out of the endolysosome for productive infection.

### GD1a stimulates transport of Py to the ER

**Figure 5:**
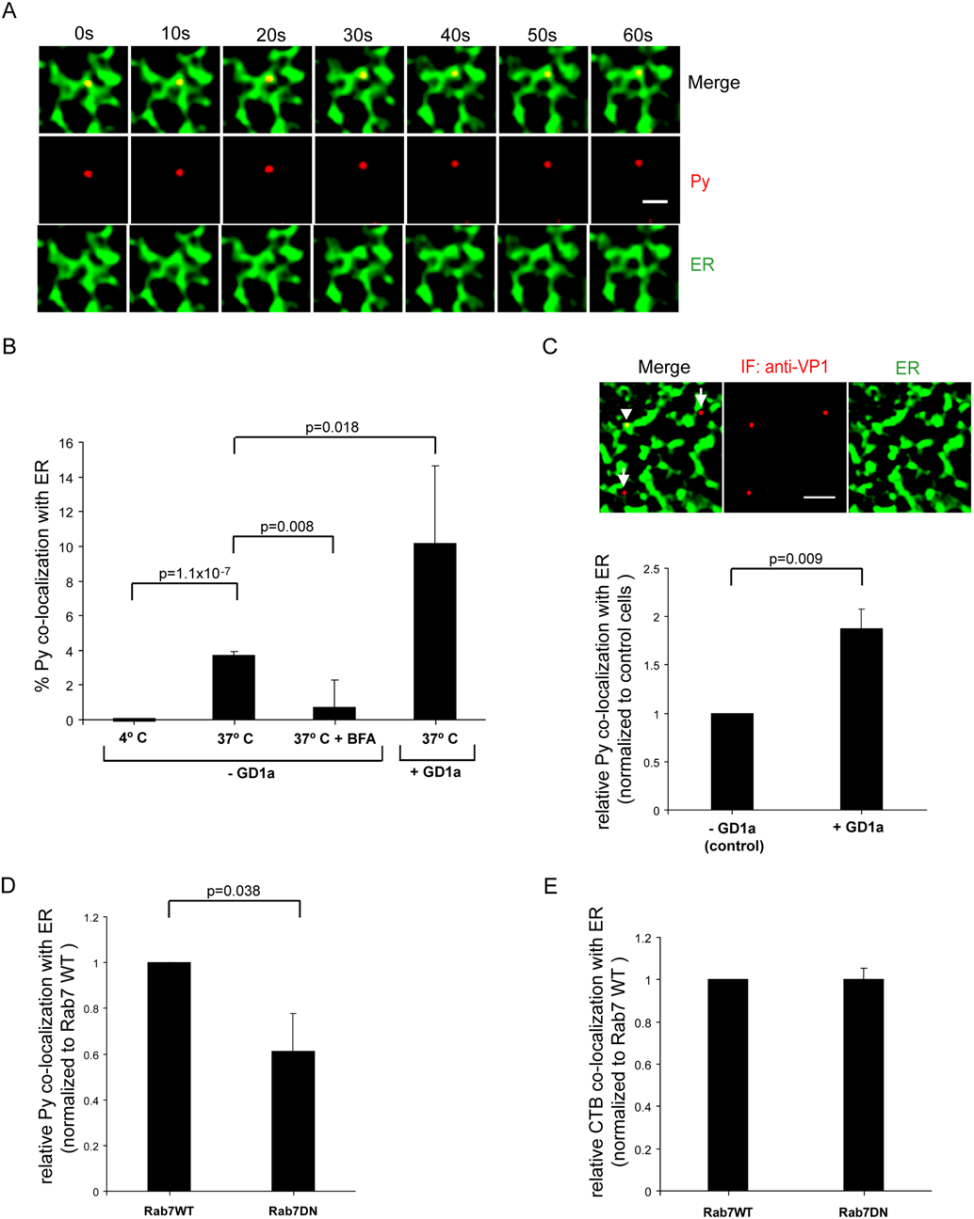
Increased co-localization of Py with the ER in GD1a-supplemented cells. (A) Live cell imaging of labeled Py co-localization with the ER. NIH 3T3 cells co-expressing CFP-HO2 and YFP-Rab7 were infected with labeled Py and the extent of co-localization of Py with the ER was analyzed 4–6 hrs post-infection. The images of the ER were subjected to filtering (see Figure S5) to more clearly define the ER tubule boundaries. Scale bar, 1 µm. (B) Quantification of Py and ER co-localization in GD1a (4°C, 37°C, BFA+37°C) and GD1a-supplemented cells. More than 300 viral particles were analyzed from at least 5 different cells. (C) Py and ER co-localization in control and GD1a-supplemented cells using immunofluorescence staining. Scale bar, 2 µm. (below) Quantification of the extent of co-localization, normalized to control cells. Arrowhead, Py that co-localized with the ER. Arrow, Py that did not co-localize with the ER. (D) Quantification of Py and ER co-localization in cells expressing either wild-type YFP-Rab7 (WT) or dominant-negative YFP-Rab7 (DN) using live cell tracking, as in A. The extent of co-localization was normalized to wild-type Rab7 expressing cells. (E) Quantification of CTB and ER co-localization in cells expressing either wild-type YFP-Rab7 or dominant-negative YFP-Rab7. Data are the mean+/−SD. A two-tailed *t* test was used.

As trafficking to the ER is required for successful Py infection [5]–[7], we tested whether the GD1a-mediated decrease in co-localization of Py with endolysosome results in increased ER localization of Py. We used two different methods to analyze co-localization of Py with the ER: live cell tracking and immunofluorescence staining. First, using the live cell tracking approach, cells co-expressing CFP-HO2 and YFP-Rab7 were supplemented with or without GD1a, and infected with labeled Py. The extent of co-localization of Py with the ER was analyzed 4–6 hrs post-infection. As the ER tubules are highly convoluted in NIH 3T3 cells, the images were filtered (Figure S5) to define the boundaries of the ER tubules and allow a more accurate analysis of Py co-localization. A typical example of a filtered image depicting co-localization of Py with the ER in real time is shown in Figure 5A. Using this method of analysis, we found that when the cells were incubated at 4°C to prevent endocytosis of the virus, no co-localization between Py and the ER was observed (Figure 5B). By contrast, an increased level of co-localization was found if the temperature was raised to 37°C (Figure 5B). At this temperature, addition of brefeldin A (BFA), a drug that blocks retrograde COPI-dependent transport between the Golgi complex and the ER, decreased the level of Py-ER co-localization. These data demonstrate that the level of co-localization observed between Py and ER at 37°C was specific and not due to random co-localization. Importantly, co-localization of Py with the ER increased in cells supplemented with GD1a when compare to non-supplemented cells (Figure 5B). This finding suggests that GD1a promotes transport of Py to the ER, consistent with a previous observation in C6 cells [9]. Moreover, by immunofluorescence staining, we found that the extent of co-localization between Py and the ER increased in GD1a-supplemented cells when compare to non-supplemented cells (Figure 5C; quantified below), similar to results from live cell tracking. These data collectively support the contention that GD1a targets Py from the endolysosome to the ER.

That GD1a caused an increase in the amount of Py which co-localized with the ER raises the possibility that trafficking of virus from the late endosome and/or lysosomes to the ER is part of the infectious pathway. This model predicts that disruption of virus trafficking to the endolysosome should hinder subsequent transport to the ER. Indeed, we found that the extent of Py co-localization with the ER decreased in cells expressing YFP-Rab7 DN when compared to YFP-Rab7 WT (Figure 5D). As a control, we show that expression of YFP-Rab7 DN does not affect transport of the non-endosomal cargo cholera toxin B subunit (CTB) to the ER (Figure 5E). We conclude that the endolysosome is an intermediate destination during the plasma membrane-to-ER transport of Py and that GD1a functions to sort the virus from the endolysosome to the ER.

### Transport of an artificial particle coated with a GD1a antibody to the ER

How does Py reach the ER? As many polyomaviruses including Py, SV40, and BK virus, and bacterial toxins such as cholera toxin, bind to gangliosides and are transported to the ER [4],[10],[26], we hypothesized that ligand interaction with gangliosides is sufficient to drive the ligand to the ER.

**Figure 6:**
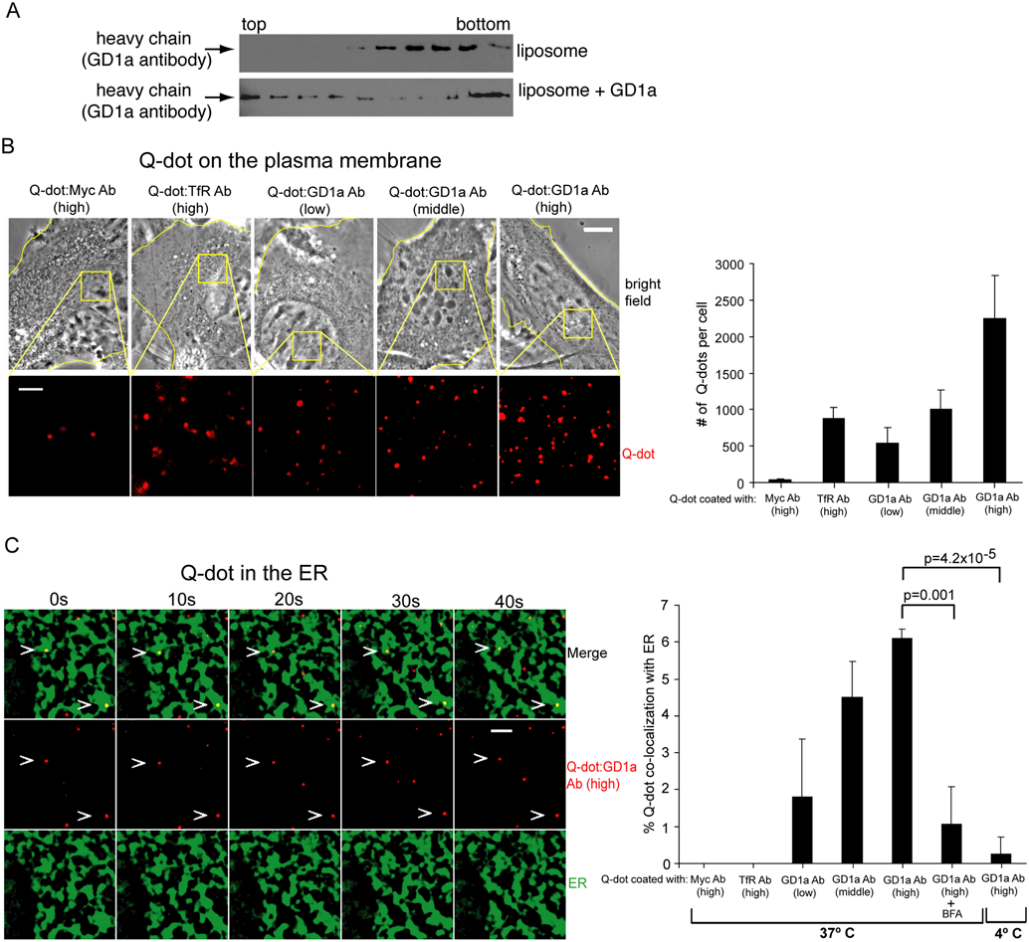
Quantum-dot coated with a GD1a antibody is transported to the ER. (A) Q-dot GD1a Ab (high) was incubated with liposomes or liposomes containing GD1a. Samples were floated in a sucrose gradient, fractionated, subjected to SDS-PAGE, and immunoblotted for the GD1a antibody heavy chain. (B) Q-dot:Myc Ab (high), Q-dot:TfR Ab (high), Q-dot:GD1a Ab (low), Q-dot:GD1a Ab (middle), and Q-dot:GD1a Ab (high) were incubated with GD1a-supplemented cells at 4°C, washed to remove unbound Q-dots, and imaged. Left panels, representative images. Yellow lines, edge of cells. Scale bars, 10 µm for bright field image, and 2 µm for Q-dot image. Right panel, quantification of the indicated Q-dot binding to the plasma membrane from at least 3 cells. Data are mean+/−SD. (C) Co-localization of Q-dot:GD1a Ab (high) with CFP-HO2 in GD1a-supplemented NIH 3T3 cells. Left panel, representative images (ER image processed as in Figure 5A). Scale bar, 2 µm. Right panel, quantification of the indicated Q-dot co-localizing with the ER at various conditions from at least 3 different cells. Data are the mean+/−SD. A two-tailed *t* test was used.

To test this possibility, we asked whether an artificial particle designed to bind GD1a in a multivalent fashion, hence mimicking a viral particle, could be transported to the ER. A fluorescent particle Quantum-dot (Q-dot) approximately 20 nm in diameter (the diameter of Py is 45 nm) was coated with either a low (0.1 mg/ml), middle (1 mg/ml), or high (10 mg/ml) concentration of a GD1a antibody (Q-dot:GD1a Ab). The ability of Q-dot:GD1a Ab particles to bind to GD1a was verified using a sucrose flotation assay used previously to demonstrate Py binding to GD1a [4]. Q-dot:GD1a Ab (high) particles were incubated with liposomes or liposomes containing purified GD1a, and the samples floated in a sucrose gradient. Fractions from the gradient were analyzed for the presence of the GD1a antibody heavy chain present on the GD1a Ab-labelled Q-dot. Flotation of Q-dot:GD1a Ab beads to the top fractions was seen with liposomes containing GD1a but not with liposomes only (Figure 6A), indicating that the Q-dot coated with GD1a antibodies can bind to GD1a gangliosides.

We then used live cell imaging to analyze the localization of various antibody-coated Q-dots to the plasma membrane and the ER in NIH 3T3 cells. Q-dot:GD1a Ab (low), Q-dot:GD1a Ab (middle), and Q-dot:GD1a Ab (high), as well as a Q-dot coated with 10 mg/ml of the control Myc (Q-dot:Myc Ab high) or 10 mg/ml of transferrin receptor (TfR) (Q-dot:TfR Ab high) antibodies, were incubated in GD1a supplemented-cells. When the Q-dots were incubated with cells at 4°C to assess plasma membrane binding, we found approximately 40 Q-dot:Myc Ab (high) and 880 Q-dot:TfR Ab (high) on the surface per cell (Figure 6B). By contrast, approximately 500 Q-dot:GD1a Ab (low), 1000 Q-dot:GD1a (middle), and 2200 Q-dot:GD1a Ab (high) bound to the surface per cell (Figure 6B). We conclude that Q-dots coated with a GD1a antibody bound to the plasma membrane in a concentration-dependent manner, presumably through interaction with GD1a on the cell surface.

Upon shifting the temperature to 37°C to allow entry, only Q-dot coated with GD1a antibody, but not Q-dot coated with Myc or TfR antibodies, can be found to co-localize with the ER (Figure 6C, left). Quantification showed that, while no Q-dot:Myc Ab (high) or Q-dot:TfR Ab (high) was found in the ER, approximately 2% of the internalized Q-dot:GD1a Ab (low), 5% of the internalized Q-dot:GD1a Ab (middle), and 6% of the internalized Q-dot:GD1a Ab (high) reached the ER (Figure 6C, right graph). It should be noted that, under the same condition, approximately 10% of the Py is targeted to the ER (Figure 5B). As a control, we found less than 1% of Q-dot:GD1a Ab (high) in the ER when the Q-dots were incubated with cells at 4°C ( a condition that prevents endocytosis) (Figure 6C, right graph). In addition, approximately 1% of Q-dot:GD1a Ab (high) co-localized with the ER when the Q-dots were incubated at 37°C in the presence of BFA (a condition that blocks cargo transport to the ER). These findings demonstrate that the extent of co-localization of Q-dot:GD1a Ab (high) with the ER detected at 37°C is not random, but specific. As an artificial particle coated with a GD1a antibody can bind to GD1a and be transported to the ER, we conclude that binding to GD1a is the fundamental mechanism for ER targeting.

## Discussion

**Figure 7:**
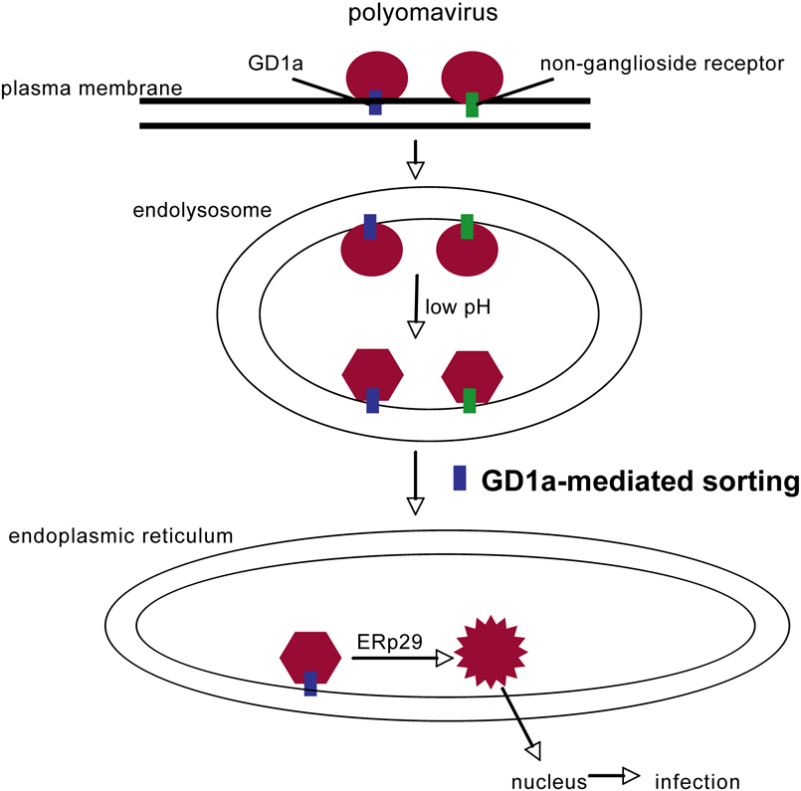
Model for sorting of Py from the plasma membrane to the ER. Polyomavirus binds to ganglioside GD1a or non-ganglioside receptors at the plasma membrane and is transport to the endolysosome. The low pH in this environment induces a conformational change on the virus that facilitates its subsequent structural alteration in the ER. Py that is bound to GD1a in the endolyosome is sorted to the ER where it undergoes an ERp29-dependent structural change [5] that initiates viral penetration across the ER membrane.

During entry, viruses must be sorted to the appropriate cellular compartments and undergo a series of conformational changes that enable them to deliver the viral genome to the cytosol or nucleus. The cellular machineries that deliver the viruses to the correct organelles for infection are poorly characterized. The murine Py is transported from the cell surface to the ER, from where it is thought to penetrate the ER membrane to access the cytosol [27] and then the nucleus to cause infection. How Py is targeted from the plasma membrane to the ER is not clear. Our results here demonstrate that Py is transported to the endolysosome where it experiences a conformational change that enhances its subsequent ER membrane penetration process (Figure 7). Importantly, we identify a novel transport pathway in which ganglioside GD1a sorts Py from the endolysosome to the ER. Finally, our findings implicate ganglioside binding as a general ER targeting mechanism. We will discuss these three points separately.

### Transport to the endolysosome

Using live cell imaging approaches, our study indicates that Py is transported through the early and late endosomes, finally reaching the lysosome. The kinetic analysis suggested that the rate of Py transport to the endosomal compartments is significantly slower than the rate observed for classic ligand-receptor complexes, such as the low-density lipoprotein (LDL)-LDL receptor and transferrin-transferrin receptor complexes. For instance, whereas Py begins to accumulate in the late endosome and lysosome 0.5–2 hrs post-infection, the entire process of delivering the LDL-LDL receptor complex to the late endosome (where LDL is released from the receptor) followed by recycling of the receptor to the plasma membrane is accomplished in 12 min [28]. Similarly, endocytosis of the transferrin-transferrin receptor complex to the late endosome and recycling of this complex to the cell surface is completed in 16 min [29]. Factors that account for the slow rate of Py internalization are presently unclear. A previous study suggested that transport of ganglioside from the cell surface to the lysosome takes place in approximately 10–15 minutes [30]. Thus it is unlikely that the intrinsic rate of ganglioside transport to the endolysosome accounts for Py's slow internalization into this compartment. Instead, it is likely that the relatively large size of the viral particle hinders the transport process.

We observed that a small portion of Py remained associated with the early endosomes for a prolonged period of time. In this context, it is interesting to note that a study had shown previously the existence of two populations of early endosomes [31]. Some cargoes are transported in vesicles called the static early endosomes, while other cargoes are transported in rapidly maturing early endosomes called the dynamic early endosomes. Sorting of Py to these dynamic early endosomes may represent the infectious route, while Py trapped within the static early endosomes for an extended period of time would lead to non-productive infection.

A significant level of Py eventually reaches the late endosome and lysosome. This observation suggests that transport to these compartments provides a strategic advantage to Py during infection. Indeed, we found that the low pH in the endolysosome imparts a conformational change in Py that enables it to more efficiently undergo an ER remodeling reaction which is crucial for the ER-to-cytosol membrane penetration event [5],[25]. That blocking acidification of the endolysosome by bafilomycin A1 decreased Py infection is consistent with the results of one study [21]. However, another study found that elevating endolysosomal pH with NH_4_Cl did not affect virus infection [13], in contrast to findings that NH_4_Cl blocks virus infection in [21] and our present study (not shown). This discrepancy may be due to differences in methodology, and the high infection levels in [13] may render Py infection resistant to NH_4_Cl treatment. The nature of the low pH-induced Py conformational change is presently unknown, although it is likely irreversible as it was detected after neutralization. One possibility is that, as calcium ions have been proposed to stabilize Py structure [32], low pH-induced protonation of the Glu and Asp residues that ligate calcium ions may trigger the release of calcium, effectively destabilizing the virus.

In addition to Py, human JC and BV virus infection are also sensitive to elevation of the endolysosomal low pH [33],[34], implicating the endolysosome as part of their infection pathway. By contrast, SV40 infection is pH independent [33]. This finding is consistent with the observation that SV40 is transported to a pH-neutral compartment called the caveosome prior to reaching the ER [35]. Whether GM1, the receptor for SV40 [4], sorts SV40 from the caveosome to the ER requires further study.

### A role of GD1a in the endolysosome-to-ER transport of Py

Our data indicate that, in addition to its role in cell surface binding of Py, GD1a also plays a critical function in the intracellular trafficking of Py. As GD1a is transported normally to the endolysosome, GD1a likely carries Py from the plasma membrane to the endolysosome. However, delivery of Py to the endolysosome does not appear to be GD1a's critical function as Py can be transported to the endolysosome in ganglioside-deficient cells. This finding suggests that non-ganglioside receptors such as glycoproteins can also deliver Py to the endolysosome (Figure 7). Instead, our analysis demonstrates that the crucial role of GD1a is to sort Py from the endolysosome to the ER to facilitate infection; Py bound to non-ganglioside receptors is likely trapped in the endolysosome.

Proteins that traffic from the ER to the late endosome/lysosome normally pass through the trans Golgi. In this context, we have not observed co-localization of Py with the Golgi (not shown), similar to a previous report [9]. Whether Py-containing vesicle that is sorted out of the endolysosome fuses directly with the ER membrane or with another vesicle which then fuses with the ER membrane, is unknown.

The proposed endolysosome-to-ER transport pathway is not without precedent. For example, under a pathological condition where gangliosides in the lysosome are not degraded, these lipids are transported to the ER and induce the unfolded protein response [36]. Furthermore, during replication of the intracellular pathogen *Brucella abortus*, the *Brucella* containing vacuole which represents a phagosome fuses with the late endosomes and recruits late endosomal markers. This vacuolar maturation process is required to traffic to the ER to enable subsequent bacterial replication [37]. Interestingly, the reverse pathway in which molecules are transported from the ER to the endolysosome independent of the Golgi was recently described in the trafficking of toll-like receptors [38]. These findings collectively implicate the existence of a previously unappreciated transport pathway between the endolysosome and the ER.

It remains unclear whether GD1a sorts Py out of the early endosome or the late endosome/lysosome. Our data show that transport to the late endosome is crucial for Py infection, and that GD1a decreased Py co-localization with the late endosome and lysosome. The simplest interpretation of these findings is that Py is sorted out of the late endosome/lysosome. However, it is conceivable that a block in transport to the late endosome interfered with upstream sorting processes in the early endosome. Further analysis is required to distinguish between these two possibilities.

Lipid rafts/caveolae were shown previously to play a role in the GD1a-mediated Py infection pathway in the rat glioma C6 cells [9]. In this context, we did not detect any significant co-localization of Py with caveolae in mouse NIH 3T3 fibroblasts. Nonetheless, it remains possible that the endolysosome and the raft/caveolae-mediated pathways intersect to facilitate Py infection. In fact, recent findings demonstrate a complex crosstalk system between these two pathways traditionally viewed as independent [39],[40].

### Ganglioside binding as a general ER targeting mechanism

What might be the molecular mechanism by which GD1a sorts Py from the endolysosome to the ER? Because the 360 VP1 molecules of Py provide 360 GD1a binding sites, each virus likely binds to multiple gangliosides on the cell surface. In addition, as gangliosides are normally internalized and transported to the endolysosome, Py may recruit even more GD1a during its transport to the endolysosome. This process clusters multiple molecules of GD1a on a single viral particle.

GD1a clustering may lead to two potential sorting mechanisms. First, clustering results in the formation of a hydrophobic platform within the bilayer, which can stimulate transmembrane signaling to recruit cytoplasmic factors that mediate budding of Py-containing vesicles from the endosomal membrane. Because gangliosides are only inserted into a single leaflet, any potential transmembrane signaling would be facilitated by interactions with a transmembrane protein. Alternatively, ganglioside clustering may alter the physical properties of the membrane beneath the virus, causing membrane invagination followed by budding. This concept was recently demonstrated for shiga toxin, where toxin binding to its ceramide-based glycolipid GB3 receptor induced tubular membrane invaginations [41].

Many bacterial toxins, such as CT [26], also bind to ganglioside receptors and are transported to the ER. Their receptor-binding B subunits are often pentameric, enabling each toxin to bind to five ganglioside molecules. Thus toxin-induced clustering of gangliosides likely targets the toxin to the ER, as was recently implicated for CT [42]. We used an artificial particle designed to bind and cluster GD1a, and showed that it can be transported to the ER. This finding supports the principle that ganglioside binding and clustering provides the fundamental mechanism for ER targeting.

In conclusion, using a model murine fibroblast cell line (NIH 3T3), we have demonstrated that Py is transported to the endolysosome and then sorted to the ER by the lipid molecule GD1a to cause infection. As Py induces tumors in a variety of cell types, including cells of the mammary gland, salivary gland, and thymus [43], future experiments will clarify whether the endolysosome-to-ER pathway is observed similarly in these other cell types.

## Materials and Methods

### Materials

Purified Py, and antibodies against VP1 and large T antigen were provided by Tom Benjamin (Harvard Medical School). The CFP-Rab5a, dominant negative CFP-Rab5a (S34N), constitutively active CFP-Rab5a (Q79L), YFP-Rab7, dominant negative YFP-Rab7 (N125I) and LAMP1-YFP constructs were generous gifts from Joel Swanson (University of Michigan). The CFP-Heme Oxygenase-2 construct was from Melissa Rolls (Penn State). A monoclonal antibody against GD1a was purchased from Millipore, purified GD1a and GM1 from Matreya, monoclonal Myc antibody, Quantum dots 655, Texas Red-X, and Alexa Fluor 594 from Invitrogen, and proteinase K, trypsin, and NH_4_Cl from Sigma.

### Preparation of Texas Red or Alexa Fluor 594 labeled Py

Purified Py was labeled with Texas Red–X succinimidyl ester (1 mM) or Alexa Fluor 594 succinimidyl ester (1 mM) following the manufacturer's protocol (Invitrogen). The labeled Py was separated from excess labeling reagent using a Micro Bio-Spin 30 Column (Bio-Rad Lab).

### Preparation of Quantum dot coated with a GD1a, Myc, or TfR antibody

Quantum dot 655 (goat F (ab') 2 anti-mouse IgG conjugate) (1 µM) was incubated with a monoclonal antibody against GD1a (0.1, 1, or 10 mg/ml), Myc (10 mg/ml), or TfR (10 mg/ml) in 30 µl PBS at 4°C for 16 hrs with mixing. Protein A agarose beads were added to the sample to precipitate the excess GD1a, Myc, or TfR antibodies. GD1a-, Myc-, or TfR-coated Quantum dots were present in the resulting supernatant.

### Infection assay

NIH 3T3 cells were transfected using Effectene (Qiagen) with constructs encoding wild-type and mutant CFP-Rab5, or wild-type and mutant YFP-Rab7. 24 hrs post-transfection, cells were incubated with Py (multiplicities of infections were approximately 100 PFU/cell or 1×10^4^ particles/cell), washed after 24 hrs, and incubated for an additional 24 hrs. Cells were then fixed and subjected to immunofluorescence (IF) with an antibody against the virus-encoded large T antigen. Phase and IF images were collected with a Nikon TE2000-E microscope using the Plan Fluor Ph2 40×/Na 0.75 objective. Only those cells expressing the transfected protein were analyzed. Where indicated, GD1a (180 µM) or GM1 (180 µM) were incubated for 24 hrs prior to infection. For characterizing the effect of bafilomycin A1 and NH_4_Cl on Py infection, cells were treated with bafilomycin A1 (0.2 µM) or NH_4_Cl (50 mM) 2 hrs pre-infection, simultaneously with infection, or 3 hrs post-infection. Cells were then infected with crude Py for 3 hrs and the unbounded virus was removed by washing. The cells were incubated at 37°C for additional 48 hrs, fixed and subjected to T antigen expression analysis.

### Time-lapse live fluorescence microscopy and image analysis

NIH 3T3 cells were transfected using Effectene (Qiagen) with the indicated constructs for 1 to 2 days, and where indicated, GD1a was added 24 hrs pre-infection. Cells were incubated with labeled Py (or Q-dot) at 4°C for 0.5 hr and the unbounded virus (or Q-dot) was removed by washing. The cells were incubated at 37°C for the indicated time, and observed with a Nikon TE2000-E microscope equipped with 100× objective. Images were acquired at 5 s or 10 s intervals.

For co-localization of Py (or Q-dot) and endolysosomal markers (CFP or YFP), different color images were taken sequentially with Nikon filter cubes for Texas Red (96313), CFP (96341) and YFP (96345). For co-localization of Py with ER (CFP-HO2), the ECFP/DsRed filter set (51018, Chroma) was used to simultaneously image the two colors. The dual-color image was split to two channels by Dual-View image splitter (Optical Insight) and projected to the two halves of a CCD camera (CoolSnap EZ^2^, Photometrics). To correct the imaging mis-alignment between different channels, Py or Q-dot images were registered to the other channels by bilinear transformation. To define the boundaries of the ER clearly, the ER images were subjected to filtering with the Fast Fourier Transform Bandpass Filter embedded in Image J (NIH). The filtering settings were set to 15 pixels with large structures and up to 3 pixels with small structures, and a tolerance of direction of 5%. Co-localization was defined as overlapping of the objects of interest in the two channels for at least 30 s in a movie.

### Immunofluorescence staining

Cells were fixed with formaldehyde (3%), permeabilized with Triton X-100 (0.2%), and incubated with either an antibody against Py large T antigen or VP1. Cells were then washed, and incubated with a fluorescently tagged secondary antibody (rhodamine labeled donkey anti-rat (for large T) or anti-rabbit (for VP1).

### Cell surface binding and entry

Control or GD1a-supplemented cells were incubated at 4°C, infected with Py, and either continued to be incubated at 4°C for 1 hr or incubated at 37°C for 1 hr to allow entry. Cells were harvested and treated with proteinase K (30 µg/ml) where indicated. Proteinase K was heat-inactivated, and the lysate was subjected to SDS-PAGE followed by immunoblotting with a VP1 antibody.

### Low pH-induced Py conformational change

Py was initially incubated in phosphate buffered saline (PBS) at pH 7.5, 6.0, or 5.0 for 60 min at 37°C. Virus incubated at pH 6.0 or 5.0 were then neutralized to pH 7.5 by addition of PBS (pH 10.0). The virus was subsequently incubated with a low concentration of proteinase K (2.5 ng/ml) or a high concentration of trypsin (1 mg/ml) for 30 min at 4°C, and subjected to SDS-PAGE followed by immunoblotting with a VP1 antibody.

### ER-dependent conformational change

Py incubated at pH 7.5, or pretreated at pH 5 and neutralized to pH 7.5, was analyzed as described in [5].

### Sucrose flotation of Q-dot

Sucrose flotation analysis is described in [4], except that Q-dot coated with a GD1a antibody was used instead of Py, and a monoclonal secondary antibody fused to HRP was used during immunoblotting.

## Supporting Information

Figure S1 Morphology and distribution of early endosomes in CFP-Rab5 expressing cells, and of the late endosomes/lysosomes in YFP-Rab7 expressing cells. (A) A non-transfected cell (arrow head) and a cell expressing CFP-Rab5 (arrow) were fixed and stained with an antibody against the early endosomal marker EEA1, followed by addition of a fluorescently tagged secondary antibody. The fluorescent signal from this antibody and CFP-Rab5 are shown. (B) As in A, except cells are expressing YFP-Rab7 and an antibody against the late endosomal/lysosomal marker LAMP1 was used. Scale bar, 10 µm.
(2.79 MB TIF)

Figure S2 Lack of Py and caveolin-1 co-localization in NIH 3T3 cells. Cells expressing caveolin-1-mCitrine were incubated with Py for 20 min, fixed and stained with an antibody against Py VP1. Caveolin-1-mCitrine in green and Py in red.
(1.33 MB TIF)

Figure S3 Effect of low pH on polyomavirus conformational change. Py incubated with the indicated pH were neutralized and incubated with a high trypsin (1 mg/ml) concentration (bottom panel) or untreated (top panel). The samples were immunoblotted with an antibody against VP1.
(0.06 MB TIF)

Figure S4 GD1a does not alter the size of endolysosomal vesicles. (A) The diameters of vesicles containing CFP-Rab5 in control and GD1a-supplemented cells were measured using an automated image analysis algorithm written for Image J (NIH). The fraction of total Rab5 vesicles within indicated vesicle sizes is shown. (B) As in A, except the diameter of vesicles containing YFP-Rab7 was analyzed. (C) As in A, except the diameter of vesicles containing LAMP1-YFP was analyzed. Data are the mean+/−SD. More than 400 vesicles were analyzed from 3 cells.
(0.22 MB TIF)

Figure S5 Image filtering of the ER image. A raw image of the ER (i.e. expressing CFP-HO2) was subjected to filtering with the Fast Fourier Transform Bandpass Filter embedded in Image J (NIH), and pseudocolored. Yellow square, area used for live cell tracking in Figure 5A. Scale bar, 2 µm.
(0.68 MB TIF)
