# Supplementary figures and images for: A Lipid Receptor Sorts Polyomavirus from the Endolysosome to the Endoplasmic Reticulum to Cause Infection
Source: PLoS Pathog. 2009 Jun 5;5(6):e1000465. doi: 10.1371/journal.ppat.1000465 (PMC2685006; doi:10.1371/journal.ppat.1000465)

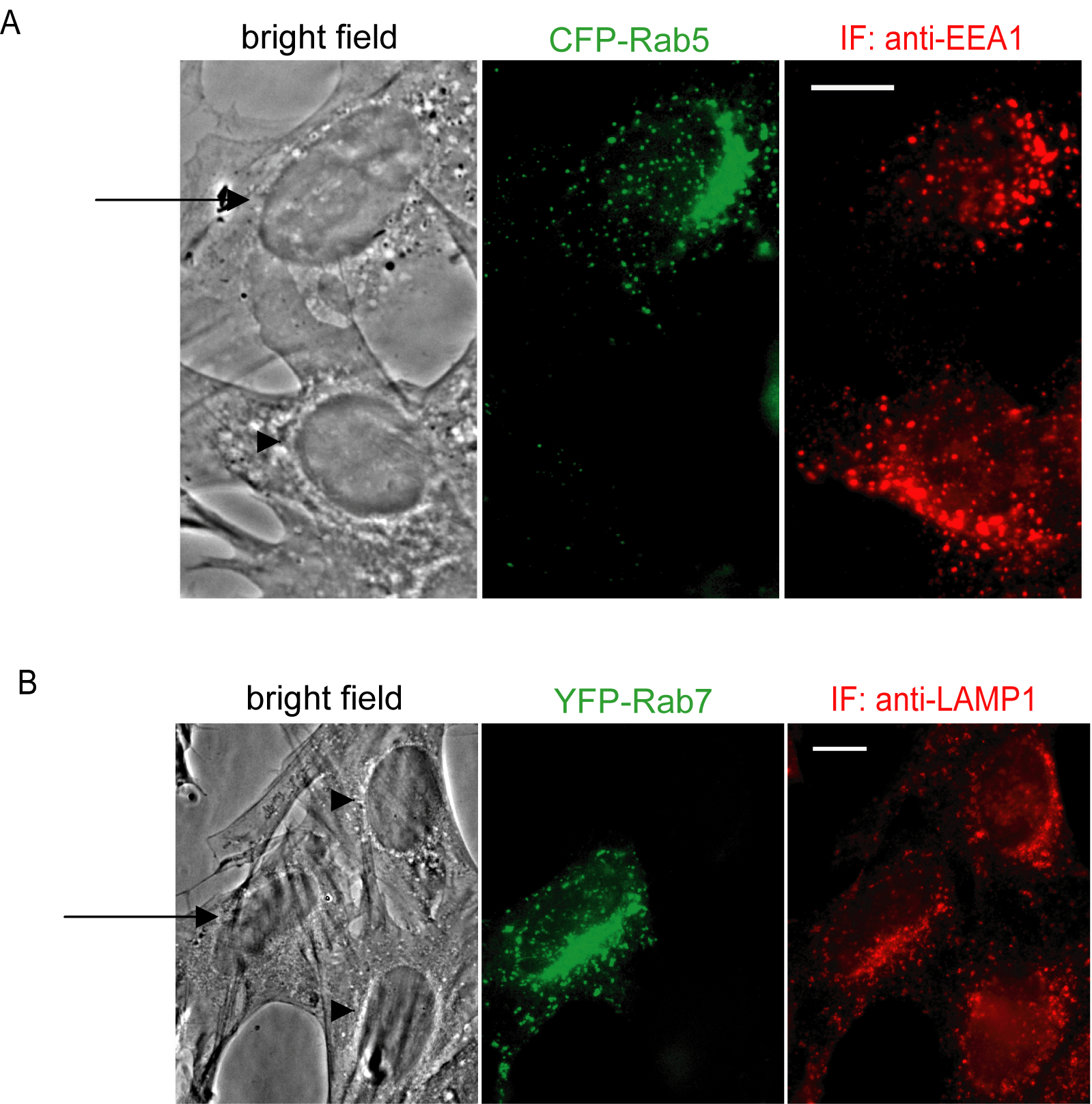

Supplement: Figure S1 — Morphology and distribution of early endosomes in CFP-Rab5 expressing cells, and of the late endosomes/lysosomes in YFP-Rab7 expressing cells. (A) A non-transfected cell (arrow head) and a cell expressing CFP-Rab5 (arrow) were fixed and stained with an antibody against the early endosomal marker EEA1, followed by addition of a fluorescently tagged secondary antibody. The fluorescent signal from this antibody and CFP-Rab5 are shown. (B) As in A, except cells are expressing YFP-Rab7 and an antibody against the late endosomal/lysosomal marker LAMP1 was used. Scale bar, 10 µm. (2.79 MB TIF) [file ppat.1000465.s001.tif]

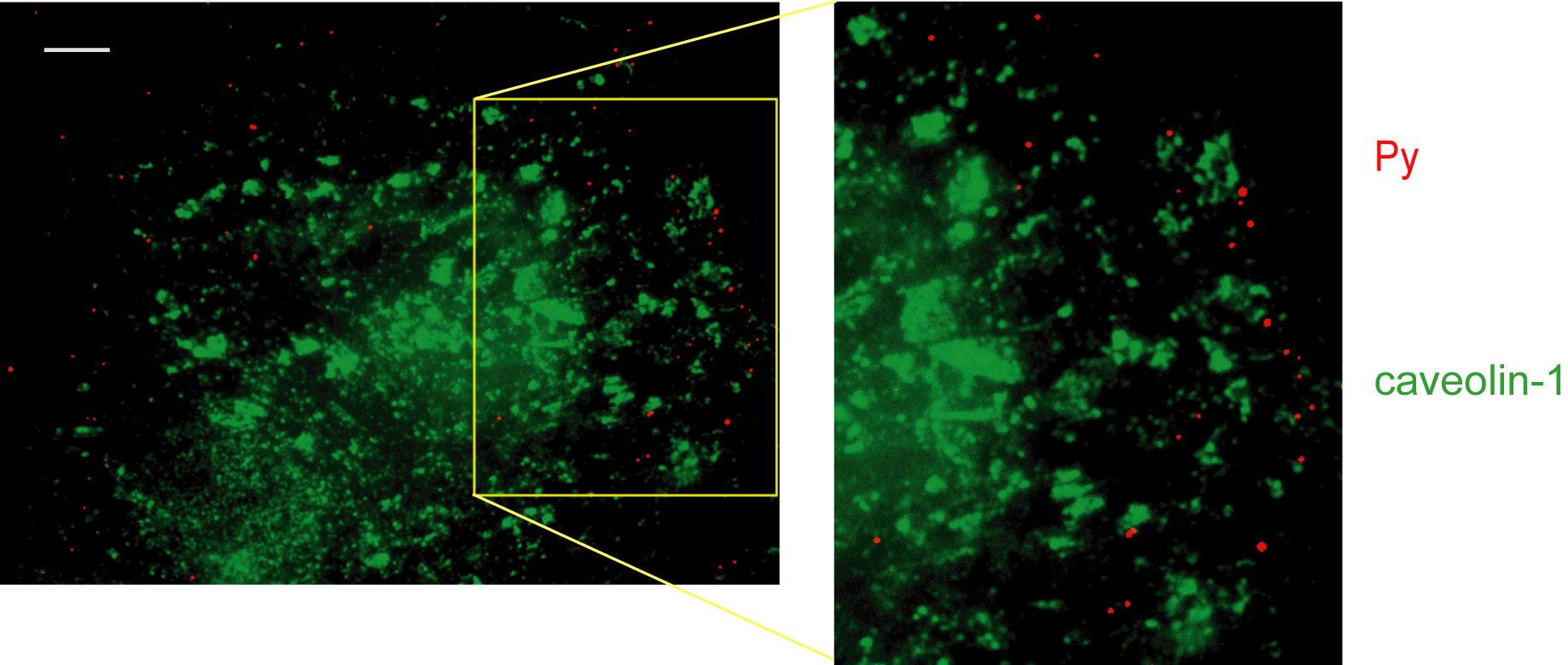

Supplement: Figure S2 — Lack of Py and caveolin-1 co-localization in NIH 3T3 cells. Cells expressing caveolin-1-mCitrine were incubated with Py for 20 min, fixed and stained with an antibody against Py VP1. Caveolin-1-mCitrine in green and Py in red. (1.33 MB TIF) [file ppat.1000465.s002.tif]

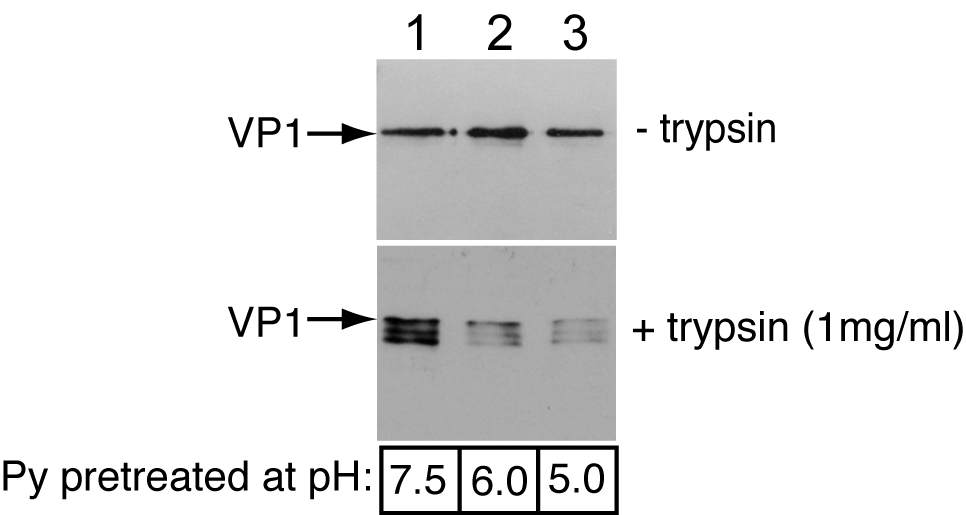

Supplement: Figure S3 — Effect of low pH on polyomavirus conformational change. Py incubated with the indicated pH were neutralized and incubated with a high trypsin (1 mg/ml) concentration (bottom panel) or untreated (top panel). The samples were immunoblotted with an antibody against VP1. (0.06 MB TIF) [file ppat.1000465.s003.tif]

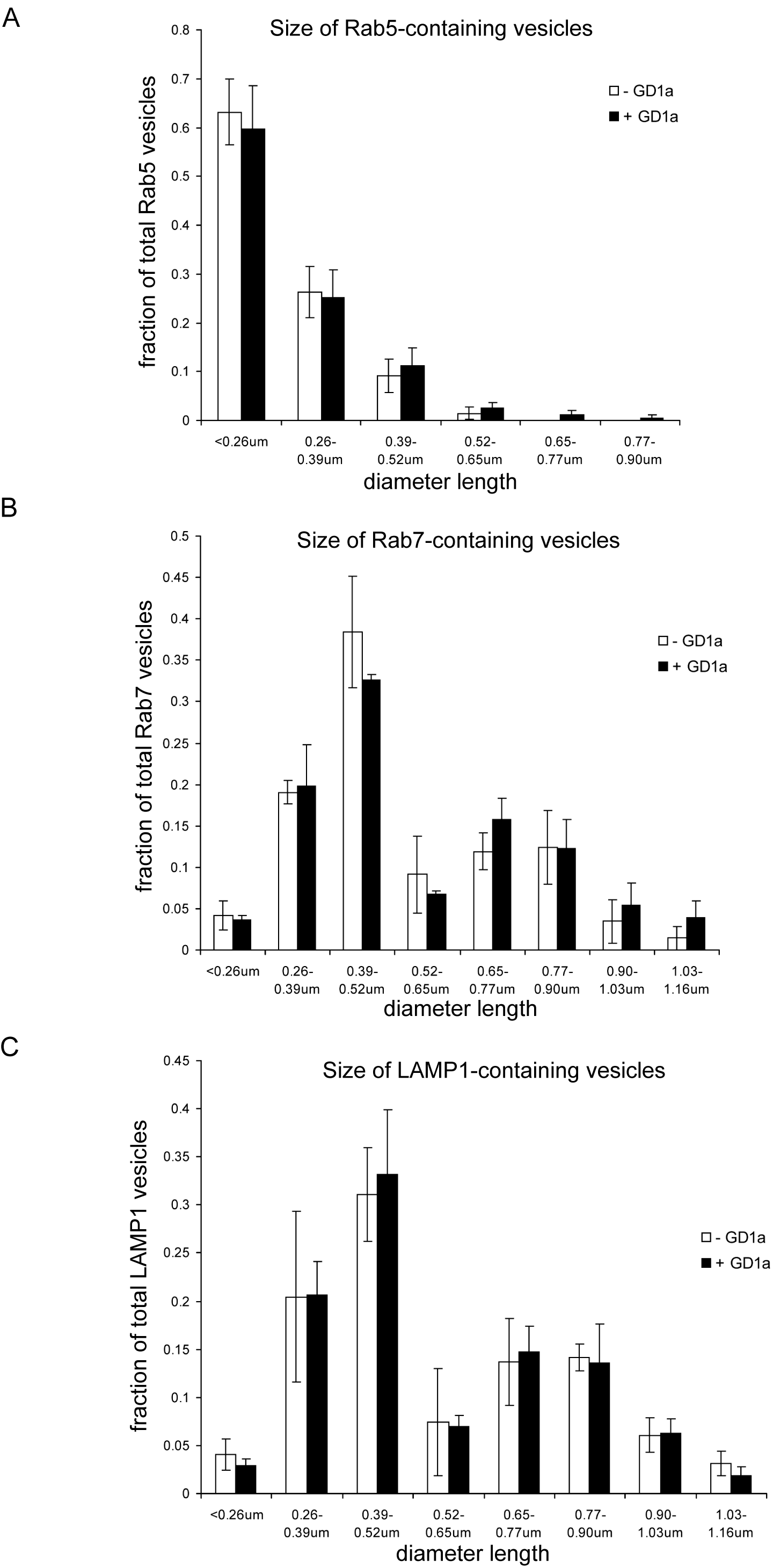

Supplement: Figure S4 — GD1a does not alter the size of endolysosomal vesicles. (A) The diameters of vesicles containing CFP-Rab5 in control and GD1a-supplemented cells were measured using an automated image analysis algorithm written for Image J (NIH). The fraction of total Rab5 vesicles within indicated vesicle sizes is shown. (B) As in A, except the diameter of vesicles containing YFP-Rab7 was analyzed. (C) As in A, except the diameter of vesicles containing LAMP1-YFP was analyzed. Data are the mean+/−SD. More than 400 vesicles were analyzed from 3 cells. (0.22 MB TIF) [file ppat.1000465.s004.tif]

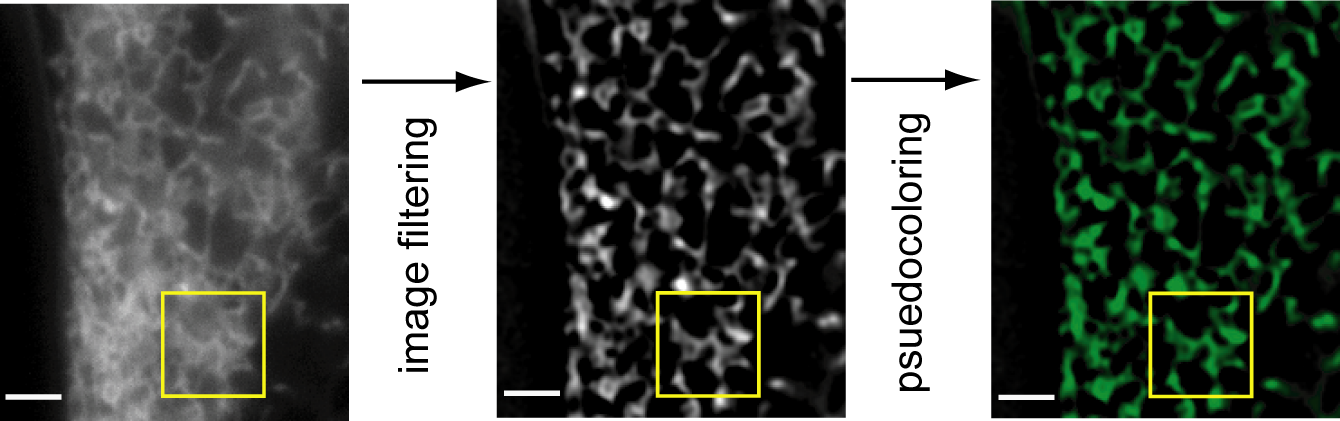

Supplement: Figure S5 — Image filtering of the ER image. A raw image of the ER (i.e. expressing CFP-HO2) was subjected to filtering with the Fast Fourier Transform Bandpass Filter embedded in Image J (NIH), and pseudocolored. Yellow square, area used for live cell tracking in Figure 5A. Scale bar, 2 µm. (0.68 MB TIF) [file ppat.1000465.s005.tif]
